# Prevalence of claims-based recurrent low back pain in a Canadian population: A secondary analysis of an administrative database

**DOI:** 10.1186/1471-2474-14-151

**Published:** 2013-04-29

**Authors:** Nicolas Beaudet, Josiane Courteau, Philippe Sarret, Alain Vanasse

**Affiliations:** 1 The PRIMUS Group, Clinical Research Centre Etienne-Lebel (CHUS), Sherbrooke, Quebec, Canada; 2 Department of Family and Emergency Medicine, Faculty of Medicine and Health Sciences, Université de Sherbrooke, 3001, 12e Avenue Nord, Sherbrooke, Quebec, J1H 5N4, Canada; 3 Department of Physiology and Biophysics, Faculty of Medicine and Health Sciences, Université de Sherbrooke, Sherbrooke, Quebec, Canada

**Keywords:** Prevalence, Recurrence, Low back pain, Administrative database, Registry, Elderly, Secondary analysis, Universal health plan

## Abstract

**Background:**

There is a vast literature reporting that the point prevalence of low back pain (LBP) is high and increasing. It is also known that a large proportion of acute LBP episodes are recurrent within 12 months. However, few studies report the annual trends in the prevalence of recurrent LBP or describe these trends according to age and sex categories.

**Methods:**

We conducted a retrospective cohort study involving 401 264 adults selected from the administrative database of physician claims for the province of Quebec, Canada. These adults, aged 18 years and over, met the criteria of having consulted a physician three times within a 365-day period between 2000 and 2007 for a LBP condition corresponding to ICD-9 codes 721, 722, 724 or 739. All data were analyzed by sex and clustered according to specific age categories.

**Results:**

We observed a decrease from 1.64% to 1.33% in the annual prevalence between 2000 and 2007 for men. This decrease in prevalence was mostly observed between 35 and 59 years of age. Older (≥65 years) women were 1.35 times more at risk to consult a physician for LBP in a recurrent manner than older men. The most frequently reported diagnosis was non-specific LBP between 2000 to 2007. During the same period, sequelae of previous back surgery and spinal stenosis were the categories with the largest increases.

**Conclusion:**

The annual prevalence of claims-based recurrent LBP progressively decreased between 2000 and 2007 for younger adults (<65 years) while older adults (≥65 years) showed an increase. Given the aging Canadian population, recurrent low back pain could have an increasing impact on the quality of life of the elderly as well as on the healthcare system.

## Background

Low back pain (LBP) is a common and costly health condition [1,2]. Lifetime prevalence has an average of 39% (+/- SD 24%), with a large variability depending on the surveyed population and the LBP definition [3]. In a lifetime, recurrent episodes will affect a large subset of the LBP population [2,4,5]. One patient out of four presenting with an acute LBP episode is likely to experience a LBP recurrence within one year [3,6]. Approximately 10% of the LBP-presenting individuals will suffer from chronic LBP [7]. These recurrent episodes and chronic cases are responsible for most of the health expenses related to LBP [8-10].

An overview of the extensive literature on low back pain led us to the following observations: 1) only 38% of prevalence studies that were reviewed provide definitions for recurrent LBP, thus making comparisons difficult [11]; 2) LBP is often reported as a point prevalence but its longitudinal progression is seldom investigated [12-14]; 3) sex and age variables are often aggregated, which limits descriptive and categorical analyses of the data.

International experts recently used a consensus approach to propose definitions for back pain [15] or recurrent low back pain [16]. This issue has been examined frequently in recent years, and the community is already adopting these standards. Regarding the prevalence of low back pain over time, the use of survey- or questionnaire-based designs is not optimal. Recall biases, small sample sizes and costs are drawbacks to longitudinal studies. While national surveys allow repeated measurements of the prevalence over time, definitions regarding low back pain are not always specific [17], and the accuracy of LBP estimates generated with large population surveys remains debatable with regards to the variations reported [18]. To complement the results obtained with surveys, the frequency of a health condition can be estimated based on the secondary analysis of administrative healthcare databases [19]. Administrative databases are often developed for preparing healthcare economic evaluations [20,21]. Recall biases are avoided [22] and data is homogeneously extracted [23]. In a universal healthcare system based on a centralized fee-for-service, the specificity of the administrative databases is high, in part because physicians promptly submit claims for the services provided to patients [24].

Therefore, by analyzing large data sets such as those found in administrative health care databases, prevalence can be determined over a longer period than in other designs. Also, the sample size is larger; therefore, categorical analyses by sex and age can be performed allowing for a better understanding of the LBP condition in different subpopulations over time.

The objective of our study was to evaluate the prevalence of claims-based recurrent low back pain in a universal health care system for the population of the province of Quebec over an 8-year period starting in 2000. By performing secondary analyses on this extensive administrative database, we provide a descriptive portrait of the longitudinal progression of the annual prevalence of recurrent LBP cases in specific age and sex categories.

## Methods

### Design and data sources

A secondary analysis of medical administrative data, obtained from the databases of the *Régie de l’ Assurance Maladie du Québec* (RAMQ), was used to perform a retrospective population-based cohort study. In the province of Quebec, the RAMQ is the government agency responsible for administering the provincial health plan, which came into effect in 1970. This plan covers medical services for Quebec residents. The RAMQ centralizes billing and service information from physicians and hospitals. In this study, the physician claims database for Quebec was used, providing information on the patient’s identification, the date of service and the primary diagnosis of the visit (four-digit International Classification of Diseases, 9^th^ Revision, or ICD-9, codes). Each Quebec resident is assigned a unique health insurance number for identification purposes, and it is further encrypted by the RAMQ for confidentiality reasons. Demographic estimates by sex and age for the province of Quebec were obtained from Statistics Canada [25]. The local Institutional Ethics Board and the *Commission d’accès à l’information du Québec* approved this study.

### Study cohort for claims-based recurrent low back pain

The cohort was selected among Quebec residents, aged 18 and older, who consulted a physician (primary care or specialist) in an ambulatory care facility (emergency, urgent care clinic or outpatient clinic) for a low back pain condition between January 1, 1999 and December 31, 2008. In this study, we considered the older population as being ageg 65 or older. 401 264 adult patients were selected using the physician claims database for “claims-based recurrent low back pain” if they had at least three identical LBP diagnoses (3-digit ICD-9 codes 721, 722, 724 or 739) within a period of 365 days, with at least one of the diagnoses in the year under study.

### Data analyses

The annual prevalence reports the proportion of patients identified with recurrent claims-based LBP for the estimated adult population in the province of Quebec for every year between 2000 and 2007. The annual frequency of ICD-9 codes was also reported according to a classification proposed by Cherkin and colleagues, which is based on clinical categories of mechanical low back problems [23]. Extraction of the data was performed with SAS (version 9.2; SAS Institute Inc, NC, USA). Graphing, linear regressions and relative risks were prepared with GraphPad (version 5.d; Graphpad Software Inc., CA, USA). Confidence intervals (99%) were determined with the Wilson score interval method.

## Results

**Figure 1:**
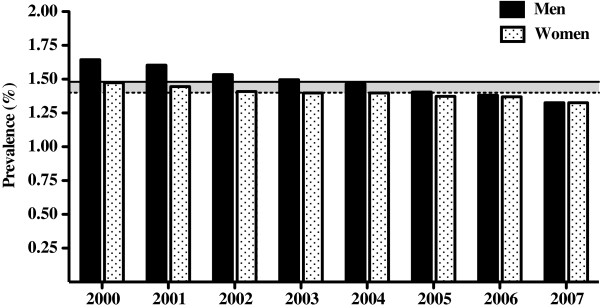
**Bars represent the annual prevalence of claims-based recurrent low back pain in the Canadian province of Quebec for men (black bars) and women (shaded bars).** The 8-year mean prevalence for men (solid line) and women (dotted line) shows the difference between the sexes (shaded area). Both distributions display a progressive and significant decrease from 2000 to 2007.

**Figure 2:**
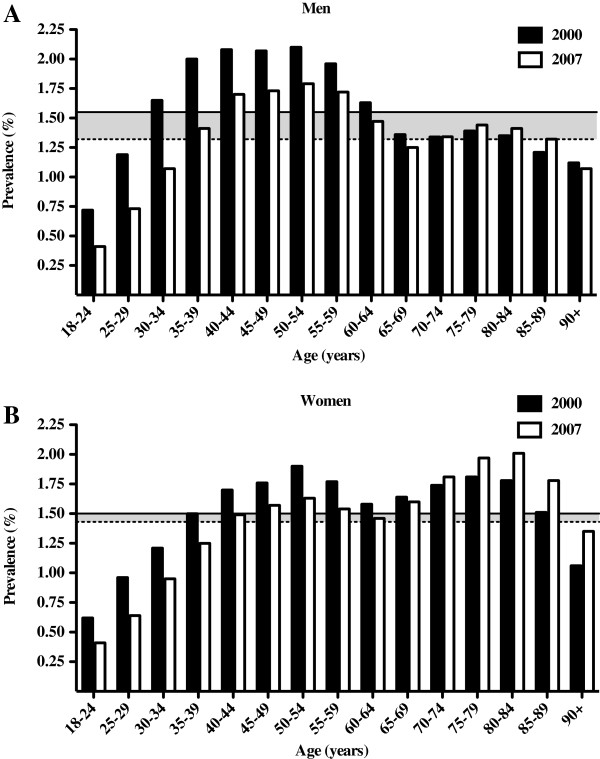
**Annual prevalence of claims-based recurrent low back pain in 2000 and 2007 by age categories and sex. A)** Profile of the male cohort prevalence. The mean prevalence in 2000 (solid line) is higher than in 2007 (dotted line). **B)** Profile of the female cohort prevalence. The mean prevalence in 2000 (solid line) is slightly lower than in 2007 (dotted line).

In 2000, among the total population of 5.8 million adults in the province of Quebec, 89 687 patients consulted a physician at least three times within a one-year period for a condition related to low back pain. This resulted in a recurrent claims-based LBP prevalence of 1.64% for men and 1.47% for women. Eight years later, in 2007, 81 329 patients were selected based on the same criteria, leading to a prevalence of 1.33% for both sexes (Figure 1). Although the demographic growth of the adult population of Quebec was on average 0.63% per year between 2000 and 2007, the prevalence of recurrent claims-based low back pain progressively decreased in the same period. A detailed analysis using 5-year age categories allowed us to determine a prevalence peak of 2.1% for adult men presenting a recurrent LBP consultation pattern between the ages of 35 to 59 in 2000 (Figures 2A). In 2007, this peak reached 1.8%, a decrease of 17%. On the other hand, in 2000, women presented a bi-phasic distribution, with peaks in their early fifties (1.9%) and in their late seventies (1.8%) (Figure 2B). Similar to men, a prevalence decrease was observed in 2007 for women under 65 years of age, while older female cohorts showed an increase (2%).

The greatest annual prevalence drop between 2000 and 2007 was observed in younger men and women (18-34 years) with a decrease of 38% and 28% respectively (Table 1). Negative slopes, reflecting the longitudinal decrease across the eight years analyzed, were also observed for all age categories corresponding to the labor force population (under 65 years). Nonetheless, even if the cohort of men, overall, showed a greater decrease in their prevalence between 2000 and 2007 than the cohort of women, men between 18 and 64 years of age were 1.10 to 1.25 times more likely than women to consult in a recurrent manner for low back pain (Table 2).

**Table 1:** Comparison of claims-based recurrent LBP prevalence by age category and sex between 2000 and 2007

**Age / Year**	**2000**	**[99% CI]**	**2007**	**[99% CI]**	**% Var**^**1**^	**Slope**^**2**^
**18-34**						
Male	1.13	[1.10 – 1.16]	0.70	[0.68 – 0.73]	−38	−0.061
Female	0.89	[0.87 – 0.92]	0.64	[0.62 – 0.66]	−28	−0.033
**35-49**						
Male	2.05	[2.01 – 2.09]	1.63	[1.59 – 1.66]	−21	−0.062
Female	1.65	[1.62 – 1.69]	1.45	[1.41 – 1.48]	−12	−0.027
**50-64**						
Male	1.93	[1.89 – 1.98]	1.68	[1.64 – 1.72]	−13	−0.032
Female	1.78	[1.73 – 1.82]	1.55	[1.52 – 1.59]	−13	−0.028
**65-80**						
Male	1.36	[1.31 – 1.41]	1.33	[1.29 – 1.38]	−2	−0.001
Female	1.72	[1.67 – 1.78]	1.79	[1.74 – 1.84]	+4	+0.016
**80 +**						
Male	1.29	[1.18 – 1.41]	1.35	[1.26 – 1.45]	+5	+0.004
Female	1.58	[1.50 – 1.67]	1.83	[1.75 – 1.91]	+16	+0.005

[1] % Var: percentage of variation between 2000 and 2007.

[2] Slope values are based on the linear regressions of the prevalence for every age category for years 2000 to 2007.

**Table 2:** Men’s relative risk for claims-based recurrent LBP compared to women in 2000 and 2007

**Age / Year**	**2000**	**[99% CI]**	**2007**	**[99% CI]**
**18-34**				
	1.26	[1.22 – 1.32]	1.10	[1.05 – 1.16]
**35-49**				
	1.24	[1.20 – 1.27]	1.12	[1.09 – 1.16]
**50-64**				
	1.09	[1.05 – 1.13]	1.08	[1.05 – 1.12]
**65-80**				
	0.79	[0.75 – 0.83]	0.75	[0.72 – 0.79]
**80 +**				
	0.81	[0.73 – 0.90]	0.74	[0.69 – 0.81]

Interestingly, there was an exception for the female cohort for which the age categories greater than 65 years were consistent with a progressive increase in the prevalence of recurrent claims-based medical visits for 2007. Based on the annual prevalence from 2000 to 2007, this increase resulted in a positive slope of 16% (Table 1). More importantly, older women were up to 1.35 times more at risk to consult a physician for low back pain than were older men, which is the reverse of the trend previously observed in adults under 65 years of age (Table 2).

Finally, the frequency of the specific diagnoses (4-digit ICD-9 codes) that were claimed by physicians in 2000 and 2007 was analyzed for all the selected patients. According to the seven-category LBP classification proposed by Cherkin and colleagues (Table 3) [23], the type of claims was rather stable with variations below 2%. As expected, the most recurrent diagnoses were part of the *non-specific back aches* category, at 66.9% and 65.1%, and the *probably degenerative changes* category, at 18.5% and 17.2%, for 2000 and 2007 respectively. In the same period, the most important increase within a diagnosis category corresponded to *sequelae of previous back surgery,* which was claimed 26 times more. *Spinal stenosis* claims also increased by 43%.

**Table 3:** Variation in the frequency of ICD-9 codes for patients with claims-based recurrent mechanical LBP in 2000 and 2007

**Condition**	**ICD-9**	**Frequency 2000 (n)**	**Frequency 2007 (n)**	**Variation over all diagnoses %**	**Variation by category % (n)**
Herniated disc	722.1	1.4%	2.0%	+0.6%	11%
	722.2	(5 270)	(5 825)		(555)
	722.7				
Probably degenerative changes	721.3	18.5%	17.2%	−1.3%	−18%
	721.5-8	(70 162)	(57 764)		(-12 398)
	721.9				
	722.5				
	722.6				
	722.9				
Spinal stenosis	721.4	1.9%	3.8%	+1.9%	43%
	724.0	(7 250)	(12 765)		(5 515)
Possible instability	724.6	0.2%	0.1%	−0.1%	−37%
		(655)	(415)		(-240)
Non-specific back aches	724.2	66.9%	65.1%	−1.8%	−14%
	724.5	(254 385)	(218 228)		(-36 157)
Sequelae of previous back surgery	722.8	0.0%	0.1%	+0.1%	2 650%
		(8)	(212)		(204)
Miscellaneous	722.3	11.2%	11.7%	+0.5%	+8%
	724.3-4	(42 422)	(39 224)		(3 198)
	724.8-9				
	739.3-4				

## Discussion

Based on the 2001 and 2006 census population counts, Quebec is the second most populous province, representing 24% of the Canadian population, and has a median age of 41.0 years. Quebec’s demographic characteristics are similar to the other Canadian provinces [26]. Canada’s public health care system offers universal coverage for comprehensive health care services, which are delivered by each of the country’s provincial and territorial insurance plans in accordance with the Canada Health Act [27]. Our secondary analysis of Quebec’s administrative claims database revealed a progressive decrease in the annual prevalence for recurrent claims-based LBP from 2000 to 2007 for both men and women. We also showed that men under 65 years of age were more likely than women to consult a physician for low back pain in a recurrent manner. This trend was reversed after the retirement age. Overall, the type of LBP diagnoses provided to patients by physicians remained stable over time, with an over-representation of non-specific backache diagnoses.

Most studies show an increase in the LBP annual prevalence over time [13,22,28] whereas few studies show a decrease in LBP diagnoses [14]. Our results show a sustained decrease in claims-based recurrent LBP prevalence from 2000 to 2007. From 1996 to 2002, Deyo and colleagues identified that the percentage of medical visits for LBP was rather stable (2.3%) [29]. In our study, unmet medical needs and accessibility issues could, in part, explain the decrease in prevalence. In fact, it was shown that the waiting time for accessing a multidisciplinary pain treatment facility was over 6 months in Canada, and that LBP was the most frequently encountered condition in these clinical facilities (28%) [30]. There is also a shortage of general practitioners in Canada, where 14% of the population reported being without a family physician in 2003 [31]. In the province of Quebec, 25% of the population reported being without a family physician in 2005 [32]. The observed decrease in the annual prevalence could also reflect an increase of complementary and alternative medicine (CAM) consultations not covered by the universal health insurance plan. A study of an American population, from 2001 to 2003, showed that among a cohort of 2000 patients, 62% were seeking care from a physician, the other 38% were receiving care from chiropractors or physiotherapists [33]. Six percent of the American population in 2002 consulted CAM practitioners for back pain [34]. Furthermore, a national survey in 2007 revealed that the most frequent condition for consulting CAM providers was back pain or back problems (17%) [35]. Chronic back pain patients are frequent users of CAM. They are thought to choose this option when their pain remains undermanaged with conventional approaches or to prevent worsening of their condition [36]. Chiropractic care was by far the preferred alternative care for chronic pain patients in a Canadian nationwide survey in 1996-1997 [36]. The administrative database used in this study did not allow us to verify these hypotheses, but future studies involving medical data linkage and private insurance records could provide answers.

### Age and sex differences

Our data showed that adult men in the age categories corresponding to the labor force represented a peak of recurrent medical visits for LBP. This is in accordance with a previous report that showed a predominance of LBP episodes in the 45-54 age category, representing 24.8% of a 15 567 patient cohort [21]. However, our results also showed that women behave differently than men with respect to the number of recurrent visits for a low back pain condition. The female cohort showed a sharper bi-phasic distribution, with a large increase in the peak of visits after the retirement age in 2007 compared to 2000. In line with this, a 2002 US national health survey on a sample of 31 000 respondents reported an increase from 15%, in the 18-24 age category, to 19.7% in the aging population (>65 years) [37]. Overall, women were also significantly more prevalent than men in that study, although the authors did not specifically state whether they were older women. Furthermore, our data also pointed out that men were at greater risk than women in age categories corresponding to the labor force. This observation was noted for early 2000, and it leveled out towards 2007. This could be the result of many factors including a transition away from professions involving manual labor in Quebec in this 8-year period, or an improvement in the prevention and security rules at work. Our results also revealed a higher prevalence for claims-based recurrent LBP in 2000 in men compared to women. However, in 2007, the prevalence of LBP in both sexes became equal at 1.33%. While many studies report a higher prevalence for chronic pain in women [38,39], a recent web-based survey study in the US revealed no difference in the prevalence between men and women for chronic LBP [7]. Also, we noted that for both sexes, there was a drop in the prevalence in the 60-64 age category in comparison to the peak in the early fifties. In Canada, the mean retirement age is 61 years of age [40], which is in line with the prevalence decrease in the 60-64 age category. It has been reported that the frequency of severe back pain increases with age, but that adults in age categories under 65 years of age are most affected by benign and mixed back pain [41]. In addition, based on a decennial survey in France, active men and women of pre-retirement age showed a higher prevalence of LBP of more than 30 days, which progressively decreased past the retirement age and stabilized around the age category of 70-74 [42]. However, our data revealed that the prevalence of claims-based recurrent LBP increased in the elderly, with older women being more at risk than older men. In older adults, the prevalence of co-morbidities increases [43]. Hypertension, lipid metabolism disorders and chronic low back pain was found to be the most prevalent triad in the elderly [44]. Also, in a Japanese national survey, a trend for a higher point prevalence of LBP complaints for women aged over 65 was reported [45]. It is noteworthy that in 2010, the population of the province of Quebec ranked among the oldest worldwide, behind Japan [45,46]. Therefore, this increase of recurrent consultations for LBP might become more frequent in aging populations, especially for older women.

### Larger proportion of non-specific LBP diagnoses

Our study confirmed that among the patients with claims-based recurrent LBP, 65% were diagnosed with non-specific LBP with respect to the classification proposed by Cherkin and colleagues [23]. This proportion is lower than the previously reported 84% [21], 76% [47] and 86% [48] for non-specific diagnoses from claims-related databases in the US, but higher than the 53% reported by Martin and colleagues based on a survey [12]. In primary care, other studies have reported that 80% of LBP diagnoses are labeled as non-specific and that no anatomopathological markers helped to explain the patient’s pain [49]. The frequency that we report here may, however, underestimate the true proportion of non-specific LBP diagnoses in the population since our cohort selection was based on the occurrence of 3 diagnoses within a 365-day period, in comparison to single LBP episodes. For instance, it was shown that a group of patients consulting physicians 6 times or more for LBP had fewer non-specific diagnoses than those consulting once (57% vs 84%, respectively) [21]. There are several hypotheses that can explain this over-representation of the non-specific label. First, in studies analyzing administrative databases, we have to keep in mind that physicians or their administrative staff may be using the most common ICD-9 codes in their specialty practice, among which are the non-specific labels. There have also been reports that among physicians, 63% felt that they lacked adequate training in chronic pain management [50]. Between 2000 and 2007, there was a substantial increase in spinal stenosis diagnoses in our analyses. Yet, a study reported that less than 50% of general practitioners were “very confident” in diagnosing specific conditions such as lumbar spinal stenosis and scoliosis in elderly populations. While diagnostic procedures are consistently focused on first identifying “red flags” or suspected serious pathologies, imaging is only recommended after 4 to 7 weeks, if no improvement in the condition is observed (Koes 2010). Therefore, patients diagnosed with a non-specific condition and initially no apparent serious pathology might have successive medical visits with a non-specific label before an exhaustive examination is conducted.

### Limitations

Our study has several limitations related to secondary analyses of administrative health care databases. First, the recent definitions for acute or recurrent LBP could not be used in the context of our study [15,16]. Indeed, the absence of clinical information on the duration and the intensity of LBP episodes in the administrative data is incompatible with the most recent definitions. Administrative data do not differentiate between a new LBP episode and a claim related to a previous episode that is ongoing [19]. The concept of recurrence in our study is therefore based only on repeatedly consulting a physician. Second, we proposed that three or more diagnoses in a period of one year would identify most of the cases of recurrent claims. A potential information bias thus remains if LBP was a secondary condition for patients presenting with multi-morbidities [51]. Indeed, physicians can only bill one diagnosis claim related to their patient’s condition, a peculiarity of the RAMQ claims database [52]. Selecting patients with only one or two LBP diagnoses could have also allowed for a greater sensitivity, but would have led to less specificity. Ultimately, a future study linking administrative and medical data could optimize both sensitivity and specificity. Time gaps between consultations have not been taken into account in this study. Time gaps between claims have been proposed as an option for improving the accuracy of the identification of the recurrence of a LBP episode. To date, the most frequently used clinical variable to report recurrence is the duration of the event [11]. The mean gap between two distinct LBP episodes was previously reported to be 40 days [19]. Algorithms involving the notion of time gaps between claims have to be further developed to obtain a more specific selection of patients in administrative databases. Medical data linkage to administrative data would also be helpful to increase the validity of such algorithms. Finally, we could not use exactly the same diagnosis categories reported by Vogt or Cherkin and colleagues [23,47], which decreased the specificity of our results. We only had access to 26 ICD-9 codes with a 4-digit precision. Fortunately, we were granted access to the most frequent LBP diagnoses reported in the literature.

## Conclusion

In summary, this study highlighted the importance of reporting LBP conditions by age categories and sex. It also showed an overall decrease of claims-based recurrent LBP prevalence in the healthcare system in recent years. Older women were more likely to consult a physician for LBP in a recurrent pattern, and this trend will probably increase in aging populations. Secondary analyses of administrative healthcare databases can be useful for determining the morbidity of prevalent health conditions as well as for surveillance, decision-making, cost-of-illness evaluations and guiding research programs.

## Competing interest

None of the authors report a conflict of interest.

## Authors’ contributions

NB participated in the design of the study, performed the analyses and drafted the manuscript. JC participated in the design and analyses. PS participated in the design. AV participated in the design. All authors read and approved the final manuscript.

## Pre-publication history

The pre-publication history for this paper can be accessed here:

http://www.biomedcentral.com/1471-2474/14/151/prepub
